# Waterborne asbestos: Good practices for surface waters analyses

**DOI:** 10.3389/fchem.2023.1104569

**Published:** 2023-01-25

**Authors:** Chiara Avataneo, Silvana Capella, Mariagrazia Luiso, Giuliana Marangoni, Manuela Lasagna, Domenico A. De Luca, Massimo Bergamini, Elena Belluso, Francesco Turci

**Affiliations:** ^1^ Department of Earth Sciences, University of Turin, Turin, Italy; ^2^ “G. Scansetti” Interdepartmental Center for Studies on Asbestos and Other Toxic Particulates, University of Turin, Turin, Italy; ^3^ RSA Srl, Società per il Risanamento e lo Sviluppo Ambientale dell’ex miniera di amianto di Balangero e Corio, Balangero, TO, Italy; ^4^ Operational Unit of Turin, Institute of Geosciences and Earth Resources (IGG), National Research Council of Italy (CNR), Turin, Italy; ^5^ Department of Chemistry, University of Turin, Turin, Italy

**Keywords:** asbestos analysis, waterborne asbestos, surface waters, guidelines, analytical methods

## Abstract

Asbestos occurrence has been mainly monitored in air so far and only limitedly considered in other matrices, such as water. Waterborne asbestos could originate from natural or anthropogenic sources, leading to non-conventional exposure scenarios. It could be a secondary source of airborne asbestos in case of water-to-air migration, particularly in case of surface moving water, such as in rivers and streams. The scarce attention dedicated to waterborne asbestos has led to a considerable fragmentation in regulatory approaches regarding the study of water samples possibly contaminated by mineral fibres. In this context, this study has been designed to test the reliability of an existing analytical method devoted to natural waters investigations. Following the operational protocol issued by the Piedmont (Italy) Environmental Protection Agency, Scanning Electron Microscopy analyses have been performed on a standard sample of waterborne chrysotile, mimicking stream water. The investigations have been performed by different operators and using different analytical setups, to verify whether the method applied has a good interlaboratory reproducibility and which could be the most error-prone analytical steps. Three data sets have been obtained on the same sample, showing a low reproducibility among each other. Possible reasons causing this discrepancy have been discussed in detail and good practices to perform reliable analyses on surface water samples containing asbestos have been proposed to help the regulatory organs to better define analytical protocols.

## 1 Introduction

Asbestos is a commercial and legislative term that indicates a group of six minerals with particular technological properties and characteristics, and dimensions which make them potentially carcinogenic when respired (length, L > 5 μm; width, W < 3 μm; aspect ratio, length to width ratio > 3:1) (WHO, 1986).

Those six minerals are chrysotile, crocidolite, and amosite (largely extracted and processed in the past to produce asbestos containing materials), anthophyllite asbestos (limitedly extracted and processed in the past due to scarce availability), tremolite asbestos and actinolite asbestos (generally occurring as contaminants in talc containing rocks) (e.g. Thives et al., 2022).

Asbestos carcinogenicity after respiration has been defined with certainty by the International Agency for Research on Cancer (IARC, 2012) and, therefore, its extraction and processing has been banned in many country (IBAS, 2022).

Since the only proved exposure route of asbestos is respiration, asbestos risk assessment is currently limited to airborne fibre monitoring in the environment (DM 06/09/1994; ANSI, 2009; ISO, 2019a; ISO, 2019b; ISO, 2019c) and occupational sites (DM 06/09/1994; NIOSH, 1994; ISO, 2014; NIOSH, 2019). Recently, other matrices have also been considered and analyses devoted to asbestos risk from contaminated soil and rocks during excavation processes were proposed (e.g., Bailey, 2020; Botta et al., 2020). In these contexts, rocks and soil could be source of secondary airborne asbestos when disturbed by human activities or meteoric events and the attention of environmental protection agencies are proposing to include new emitters and receptors in asbestos environmental risk analysis (e.g. ANSES, 2017). Furthermore, the necessity to include a wider set of minerals within the group of investigated fibres clearly emerged when the focus shifted from industrially produced to environmentally occurring asbestos (e.g. Buck et al., 2013; Erskine and Bailey, 2018; Bloise et al., 2019; Belluso et al., 2020; Berry et al., 2022).

In naturally occurring asbestos (NOA)-rich areas, mineral fibres could be released in air or water and move far from the original pollution source, possibly accumulating in rivers, lakes and sediments downwind (e.g. Jacques and Pienitz, 2022), being a possible source of secondary airborne fibres.

Mechanism of mineral fibres movement and migration through different environmental matrices (rock/soil, water, and air) is challenging and still scarcely investigated. Opposite to the migration of a molecular pollutant, the migration of a solid micrometric hazardous pollutant depends not only on site-specific characteristics, but is strongly affected by the variable characteristics of the minerals themselves, which exist in nature in a wide range of forms that differentiate the pollutant for key physio-chemical characteristics, including morphology of the particles, solubility, surface charge, and mechanical properties (Schreier, 1989).

One of the less investigated yet deeply concerning scenario is the release and migration of asbestos in natural waters (surface waters and groundwater). Waterborne asbestos has been detected in several surface waters of former mine areas (Kashansky and Slyshkina, 2002; Anastasiadou and Gidarakos, 2007; Koumantakis et al., 2009; Turci et al., 2016; RSA, 2019). On the other hand, few available studies reported the presence of asbestos or other mineral fibres in the groundwater system (Buzio et al., 2000; Avataneo et al., 2021).

Environmental migration of asbestos below ground has been only recently investigated and the movement of waterborne chrysotile has been attested in sandy porous media when enhanced by dissolved organic matter (Mohanty et al., 2021).

Since waterborne asbestos has not been routinely monitored in the past, it could be considered as an Emerging Pollutant (EP) in the water matrix (Avataneo et al., 2022), leading to non-conventional exposure ways. Indeed, waterborne asbestos could be a secondary source of airborne asbestos linked to possible water-to-air migration, particularly relevant considering surface moving water, such as in rivers and streams. Fibres can be released in air under collapse of bubbles and foams from polluted waters in natural environment.

The water-to-air migration could be particularly relevant to human health if polluted waters are used indoor, where wet/dry cycles in showers or humidifier systems might cause an abundant release of airborne fibres, as reported by Roccaro and Vagliasindi, (2018). On the contrary, other studies regarding the use of contaminated tap water in houses (Webber et al., 1988) or dealing with the use of drum-type humidifier (Meranger et al., 1979) reported negligible amount of airborne asbestos released indoor.

Waterborne asbestos could also be ingested, especially if present in tap water. Up to date, possible asbestos carcinogenicity by ingestion still remains unclear and not scientifically proven (WHO, 2021), while the danger linked to asbestos respiration is well documented, as already reported.

The authors believe that greater attention should be dedicated to waterborne asbestos since natural water is largely exploited for agricultural and industrial processes and as a source of drinking water. Indeed, asbestos occurrence in water could pose a generally underestimated risk for locally resident general population and the environment. The use of contaminated water for fields watering could even cause plant growth stress, as previously attested (Schreier, 1989).

The past limited attention to waterborne asbestos occurrence has led to a considerable fragmentation regarding the study of water samples and a lack in regulatory approaches and monitoring.

Limited number of standardised methodologies for the preparation and analyses of waterborne asbestos exists today and they mainly concern drinking water. A recent publication has been issued containing detailed description of available methods for waterborne asbestos analyses (Malinconico et al., 2022).

Particularly, the United States Environmental Protection Agency (US-EPA) issued a method for the determination of asbestos fibres over 10 µm in length in drinking water, requiring investigations by means of Transmission Electron Microscopy coupled with Energy Dispersive Spectroscopy (TEM-EDS) (US-EPA, 1994), and also defined a maximum contaminant level (MCL) of asbestos in drinking water of 7⋅10^6^ fibres per litre [f/L] (US-EPA, 2004), considering only fibres longer than 10 µm. Significantly, the current legislation concerning water quality set by the European Parliament and Council (2020) does not contain limit values for asbestos in drinking water.

In Italy, where this study has developed, an MCL of asbestos in drinking water has not yet been enforced and few analytical protocols are made available by national authorities for the environmental protection and health. The ISS.EAA.000 method by the Italian National Institute of Health (ISS, 2016) is an analytical protocol for the determination of asbestos concentration in drinking water samples, based on Scanning Electron Microscopy coupled with EDS (SEM-EDS) analyses. Fibres with length greater than 5 µm and aspect ratio greater than three are considered, according to the WHO criteria for respirable fibres (WHO, 1986). Fibrous structures showing width greater than 3 µm are counted as well, despite the WHO airborne counting guidance. Dimensions and morphological characteristics (e.g. fibre, bundle, and aggregate) for every counted structure have to be recorded.

Another similar method is available in Italy, the U.RP.M842 rev.03 operating method (ARPA Piemonte, 2016) and its later revision rev.05 (ARPA Piemonte, 2021) issued by the Piedmont Environmental Protection Agency, which involves again SEM-EDS analyses. This second method applies to general water samples, not just drinking ones.

In addition to this scenario, it is important to underline that supervisory authorities in Italy have not yet enforced official inter laboratory circuits regarding waterborne asbestos analysis. Only few unofficial round-robin tests exist led by private companies.

It is generally accepted that methodologies that rely on microscopy examination are strongly affected by the analyst’s experience and his/her ability to correctly discriminate between asbestos and non-asbestos minerals (Cossio et al., 2018). In the case of waterborne asbestos measurements, the incomplete or poorly disseminated methodologies and the consequent lack of official interlaboratory round-robin routines, further worsen the variability and undermine the reliability of the analytical measures.

In the framework of a highly fragmented methodological scenario, it is fundamental to evaluate analytical instrumental setups and methodologies to verify if available procedures are reliable and give good interlaboratory reproducibility, in order to perform a reliable asbestos quantification in water samples.

To this aim, a study has been designed to define good practices for the quantification of waterborne asbestos, particularly in surface water samples. To define these important aspects, the ARPA Piemonte operating method U.RP.M842 rev.03 (ARPA Piemonte, 2016) has been applied to one standard polluted water sample created *ad hoc* by suspending well characterised chrysotile in tap water, mimicking contaminated surface water of streams. Two independent laboratories and several analysts participated in the study on waterborne asbestos to verify the interlaboratory reproducibility of the adopted method and sort out the most error-prone operational steps in the sample analysis.

Principal aspects and good practices to perform reliable analyses will be described and discussed to define common guidelines for waterborne asbestos study. An attempt will be made to identify and consequently avoid the error-prone steps during the analyses and data elaboration.

## 2 Materials and methods

### 2.1 Sample

Chrysotile asbestos standard from Balangero former mine (Italy) was suspended in conventional tap water and magnetic stirred for about 1 h to achieve suspension homogeneity. Water was filtered on 0.22 µm pore membranes and examined to exclude asbestos contamination prior use.

The chrysotile standard is a powder originating from the Balangero former mine, located next to Turin, Italy (45°17′32″N 7°31′01″E) (e.g. Fornasini et al., 2022), provided by RSA Srl^1^, the public company in charge of the remediation and environmental development of the former asbestos mine site of Balangero and Corio municipalities.

More specifically, chrysotile used to prepare the sample is a 1:1 mixture of the medium-to-long (“Class 5mx”, after RSA Srl classification) and short fibres (“Filler”, after RSA Srl classification), partially processed chrysotile coming from the former mine industry, therefore previously selected for its mineralogical purity. To promote the disaggregation of fibre bundles and mixing, the two chrysotile products were gently crushed in acetone using an agate mortar.

An amount of the chrysotile water suspension is then added to a 38.28 L tap water tank to obtain the analytical sample. The tank is equipped by two pumps and a bubbler that are activated to homogenise the suspension. The apparatus is placed in a close system to avoid ambient air dispersion of fibres.

After 1 h of water moving and stirring, mimicking water motion in streams, one water sample is collected from the tank and prepared following the ARPA Piemonte U.RP.M842 rev.03 operating method (ARPA Piemonte, 2016).

Based on the chrysotile quantity added to the water in the tank, the nominal waterborne concentration is 137.40 μg/L and, therefore, the prepared sample has a high chrysotile content. This high concentration sample, expected to contain a great number of fibres, was considered to give statistically sound data in term of concentration calculation. The prepared water sample was intended to represent the worst-case scenario that could be found in contaminated surface waters, like in moving streams of the Balangero former mine area.

Other samples containing nil, low and mid chrysotile concentration were prepared and analysed (see Supplementary Table S1), but they are not considered in this study for the very low chrysotile content detected in water.

### 2.2 Sample preparation for analysis

The water sample was prepared following the ARPA Piemonte U.RP.M842 rev.03 operating method (ARPA Piemonte, 2016), filtering an aliquot of the chrysotile water suspension on a 47 mm diameter Polycarbonate (PC) porous membrane with 0.8 µm pore diameter.

A volume of 0.05 L was filtered to avoid clogging the filtration system.

### 2.3 Sample analysis

Membrane obtained by filtration was let to dry at room temperature and then cut in 3 parts. The first portion (membrane portion n.1) was coated by a thin gold layer to make it conductive and then was analysed by an operator in Lab 1 using a TESCAN VEGA 3 SBH Vega TC ver. 4.2.25.1 SEM coupled with an INCA microanalysis suite EDS, Oxford Instruments.

The second and third portions were analysed by another operator in Lab 2 using a JEOL JSM IT300LV SEM coupled with EDS detector Oxford INCA Energy 200, INCA X-act SDD thin window for analyses. Membrane portion n.2 was coated by graphite layer, while membrane portion n.3 was coated by gold.

All analyses were carried out following the ARPA Piemonte U.RP.M842 rev.03 operating method (ARPA Piemonte, 2016): after checking particles distribution uniformity on the membrane portion at low magnification, an area of up to 1 mm^2^ is scanned acquiring micrographs at 4000X and all the fibrous structures are analysed. EDS semi-quantitative spectra are acquired on all detected fibres. Chemical analyses allow, together with morphology, to define whether the fibre detected belongs to the asbestos group. If so, the fibre is counted and measured.

The operating method U.RP.M842 rev.05 (ARPA Piemonte, 2021) was not used in the analytical phase because it was not yet issued at the time of the study.

Technical details about instrumental conditions, sample preparation and image resolution are reported in Table 1.

**TABLE 1:** Instrumental conditions used in the study to perform waterborne asbestos analysis.

Membrane portion number	1	2	3
Analytical laboratory	Lab 1	Lab 2	Lab 2
Electron beam energy	20 kV	15 kV	15 kV
Emitter	W filament	W filament	W filament
Metal layer for conductivity	gold	graphite	gold
Image resolution	15 pixel/µm	32 pixel/µm	32 pixel/µm
Minimum visible detail	0.067 µm	0.031 µm	0.031 µm

If fibres are found in high amount, the count could stop when 100 fibres are counted and at least 20 microscopic fields (areas displayed on the used screen at the work magnification) are observed, according to the U.RP.M842 rev.05 operating protocol (ARPA Piemonte, 2021). No indications concerning the image acquisition mode (secondary electron-SE- or backscattered electrons-BSE) are reported in the operating protocol. In this study SE images are generally acquired and a few BSE micrographs are acquired to compare with SE images.

They are considered as fibres all the particles falling in the WHO (1986) criteria, therefore having length (L) > 5 μm, width (W) < 3 µm and aspect ratio (length/width) > 3:1. Bundles with W ≥ 3 µm are included in the count. For this study, shorter structures (L ≤ 5 µm) were counted and two different concentration values (considering only respirable WHO fibres or all fibres) have been calculated.

The following *counting criteria* were applied:

1) a fibre whose both ends are visible within the micrograph is counted as 1;
2) a fibre whose only one end is visible within the micrograph is counted as ½;
3) a fibre whose both ends are outside the border of the micrograph is not counted;
4) bundles in which it is not possible to distinguish different fibres are counted as 1;
5) the occurrence of aggregates has to be documented, if fibres are sufficiently separated they have to be counted;
6) if a fibre is partially covered by a particle, it has to be counted and the visible portion measured.

The final fibres concentration in number of fibres per litre [f/L] is calculated following the formula:

$$C=\frac{A_{TOT}∙f}{A_{A}∙V},$$ (1)

Where:

$A_{TOT}$ is the total membrane area on which filtration was made [mm^2^]; *f* is the total number or fibres counted; $A_{A}$ is the analysed area of the membrane [mm^2^]; *V* is the water volume filtered through the porous membrane [L].

All concentration data are provided with lower fiducial limit (LFL) and upper fiducial limit (UFL) which represent the 95% confidence limit, based on the hypothesis of a Poisson distribution of fibres on the membrane.

For Lab 1 the analytical method, given the instrumental characteristics and the experimental conditions used, has a limit of detection (LOD) of 6.17·10^5^ f/L for waterborne chrysotile.

For Lab 2 the analytical method has a LOD of 6.76·10^5^ f/L for waterborne chrysotile.

Both Lab 1 and Lab 2 participated to unofficial round-robin interlaboratory circuits devoted to waterborne asbestos investigations. Lab 1 always scored positive evaluations, while Lab 2 investigations resulted in a slight underestimation of waterborne asbestos content in one circuit.

In addition to the evaluation reported in the operating method followed (ARPA Piemonte, 2016), it is possible to add a step in the analytical process, expressing the concentration in mass per litre [µg/L]. Starting from dimensions measurement, the volume of each detected fibre is calculated approximating it to a cylinder and then the mass is calculated by multiplying volume by density (2.6 g/cm^3^ for chrysotile).

Therefore, fibres concentration in µg/L is calculated as follows:

$$C=\frac{A_{TOT}∙m_{c}}{A_{A}∙V},$$ (2)

Where:

$A_{TOT}$ is the total membrane area on which filtration was made [mm^2^]; $m_{c}$ is the chrysotile mass found [µg]; $A_{A}$ is the analysed area of the membrane [mm^2^]; *V* is the water volume filtered through the porous membrane [L].

This approach is borrowed from the Italian method to evaluate asbestos in massive samples (DM 06/09/1994, All.1B). Here is also reported the method to calculate the error referring to mass concentration, which is expressed as a ΔC on the concentration (C ± ∆C).

ΔC in µg/L is calculated as follows:

$$∆C=\left(\frac{1}{\sqrt{N}}+\frac{\sqrt{\frac{\underset{i}{\sum }{\left(f_{ave}-f_{i}\right)}^{2}}{N∙\left(N-1\right)}}}{f_{ave}}\right)∙C,$$ (3)

Where:

*N* is the number of fibres found in the analysed area; $f_{ave}$ is the average mass of fibres [µg]; $f_{i}$ is the mass of the *i^th^* fibre [µg].

The analytical method used by Lab 1, given the instrumental characteristics and the experimental conditions used, has a LOD of 0.7 μg/L for waterborne chrysotile.

For Lab 2, the analytical method has a LOD of 1.7 μg/L for waterborne chrysotile.

## 3 Results

Data regarding waterborne chrysotile concentration in the sample are reported in Table 2 and expressed both in f/L and µg/L. A comparison has been made among data obtained by Lab 1 and Lab 2, that also carried out analyses using two different instrumental conditions (see Table 1). The three datasets will be hereafter named Lab 1_Au layer, Lab 2_C layer and Lab 2_Au layer, depending on the analytical laboratory which made the analysis and the metal coating used that produced the data. For sake of clarity, we want to remark that the three replicate analyses refer to measurements that were carried out on different portions of the same membrane. An area of about 0.1 mm^2^, correspondent to a minimum of 20 microscopic fields of view, was observed due to the high number of fibres found, according to the U.RP.M842 rev.05 operating protocol (ARPA Piemonte, 2021). All fibres detected were classified as chrysotile, based on EDS semi-quantitative spectra acquired.

**TABLE 2:** Experimental data obtained on the sample, using different setups. The confidence limits (LFL-UFL and ΔC) were calculated only for the respirable fibres count, following the methodologies reported in the text.

		Lab 1_Au layer	Lab 2_C layer	Lab 2_Au layer
Respirable WHO fibres	Number of fibres evaluated (L > 5 µm)	228	126	142
	Concentration [f/L]	4.44·10^7^	2.85·10^7^	3.21·10^7^
	LFL	4.13·10^7^	2.49·10^7^	2.66·10^7^
	UFL	4.76·10^7^	3.54·10^7^	3.74·10^7^
	Concentration [µg/L]	39.49	103.43	66.60
	ΔC [µg/L]	18.48	25.93	15.62
	Average mass of a fibre [µg]	8.91 10^−7^	3.63 10^−6^	2.08 10^−6^
All fibres	Total number of fibres (all lengths)	241	184	183
	Concentration [f/L]	4.97·10^7^	4.16·10^7^	4.14·10^7^
	Concentration [µg/L]	39.63	107.22	77.60
	Average mass of a fibre [µg]	8.45 10^−7^	2.58 10^−6^	1.87 10^−6^
	% respirable fibres	94.6%	68.5%	77.6%
	% fibres W < 0.2 µm	95%	59%	50%
	Number of bundles (W > 0.5 µm)	3	4	4
	Mean fibre aspect ratio	168.4	70.2	74.5

Regarding respirable fibres (WHO, 1986), concentration values in f/L are provided with relative lower and upper fiducial limit (Table 2), as indicated in the operating method used (ARPA Piemonte, 2016) designed for water samples. On the contrary, data in µg/L are provided with an error range derived from the methodology defined for massive samples (DM 06/09/1994, All.1B), since the Italian method for waterborne fibres does not contain mass concentration calculation.

Concentration values in f/L obtained by Lab 1 (both counting all fibres or just respirable ones) are always greater than those found by Lab 2, particularly whether only fibres with L > 5 µm are considered. Waterborne chrysotile concentration calculated considering only respirable fibres was 4.44·10^7^ f/L for Lab 1_Au layer against 2.85·10^7^ f/L for Lab 2_C layer or 3.21·10^7^ f/L for Lab 2_Au layer. Even considering the fiducial limit ranges (LFL-UFL), Lab 1 results do not overlap with those of Lab 2.

Regarding only the two sets of values provided by Lab 2, the results in f/L considering respirable fibres are comparable and the fiducial limit are well overlapping. The correspondence between the concentration calculated on C coated and Au coated membrane portions by Lab 2 is even better when counting all fibres.

The misfit between the three data sets in f/L is particularly evident when only respirable fibres are considered, because Lab 1 was able to see a greater number of fibres. Indeed, in this case Lab 1 detected 228 fibres against 126 detected by Lab 2 on sample coated by C layer or 142 on Au coated sample. If all fibres were counted (even shorter than 5 µm) the reproducibility between the three measurements would have been better (241 against 184 or 183), producing more similar concentration values (4.97·10^7^ f/L for Lab 1, 4.16·10^7^ or 4.14·10^7^ f/L for Lab 2_C layer or Lab 2_Au layer, respectively).

**FIGURE 1:**
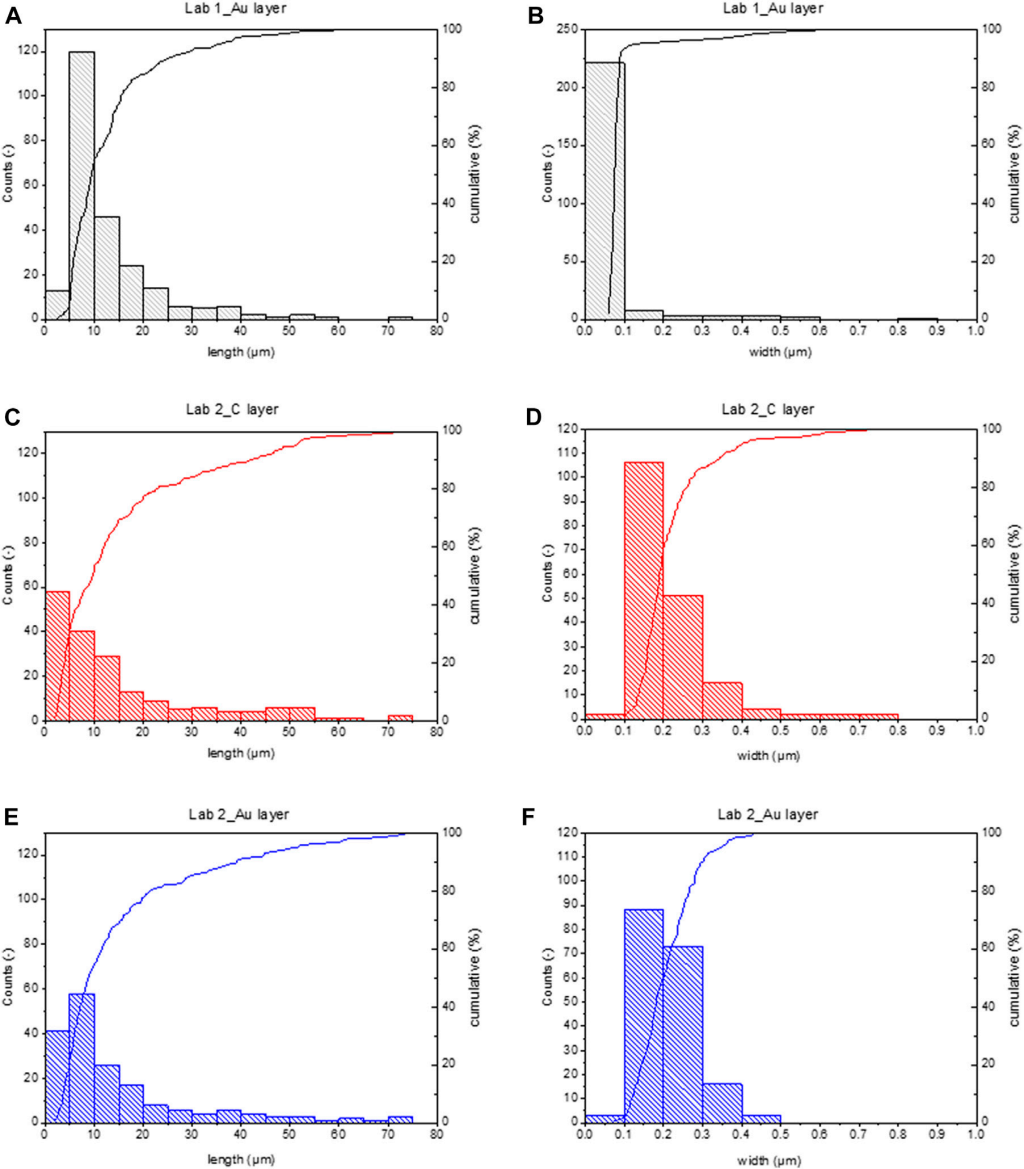
Histograms of particle size distribution and cumulative curve calculated measuring fibres in the analysed water sample, using different setups. All fibres are considered (even shorter than 5 µm). **(A)** length and **(B)** width distributions calculated on Lab 1_Au layer data. **(C)** length and **(D)** width distributions calculated on Lab 2_C layer data. **(E)** length and **(F)** width distributions calculated on Lab 2_Au layer data.

This gap is generated because Lab 1 can see more fibres, particularly thin fibres. As reported in Table 2, 95% of fibres detected by Lab 1 are thinner than 0.2 µm, while only 59% or 50% of fibres detected by Lab 1 are thinner than 0.2 µm. This is confirmed by particle size distribution regarding width reported in Figures 1B, D, F. Almost all fibres detected by Lab 1 show a width smaller than the minimum width detected by Lab 2.

Indeed, width ranges are not overlapping, particularly regarding the lower limit. Lab 1 could detect fibres down to 0.06 µm in width (comparable with the minimum detail visible with this instrumental setup), while the width limit for Lab 2 was around 0.1 µm, even if the theoretical minimum detail limit was 0.031 µm (see Table 1). As a consequence, Lab 1 width distribution shows a median <0.1 µm, corresponding to the 50% of the cumulative curve, while the median value in Lab 2 distributions is more than the double, around 0.2 µm. Comparing particle size distributions in Lab 2_C layer and Lab 2_Au layer (Figures 1D, F) distributions are similar despite the fact that in C layer larger fibres were found, up to 0.8 µm width.

Concerning length, Lab 2 counted a greater number of short fibres (respirable fibres are 68.5% or 77.6% on total), particularly in the membrane portion coated by C layer (Figure 1C). Regardless, the length ranges and particle size distributions detected in different membrane portions are comparable, showing a median value at around 10 µm and a maximum at around 70 µm (see Figures 1A, C, E).

The difference in size and morphology of fibres detected by the two laboratories is confirmed by the different aspect ratio values (length/width) (Table 2), indicating that Lab 1 could see thinner and longer fibres.

Concerning values in mass, the trend is inversed: concentration values are higher for Lab 2 even if they counted less fibres. This is because, in general, they saw bigger fibres (particularly in terms of width). This is also confirmed by the average fibre mass, which is one order of magnitude higher for measurements made by Lab 2 than Lab 1.

Bundle occurrence in the filter portions analysed by Lab 2 (four against three found by Lab 1) could contribute to increase the concentration result in mass per litre. It is important to underline that all bundles detected have dimensions falling in the respirable limits, thus they were counted as single fibre where it was not possible to recognise fibres forming the bundle.

If only respirable fibres are considered, the values obtained on Lab 2_Au layer membrane are always middle way between the concentration calculated by Lab 1_Au layer or on Lab 2_C layer, for concentration calculated in both number or mass per litre.

## 4 Discussion

Some of us (Avataneo et al., 2022) have recently demonstrated that high level of waterborne chrysotile can generate an airborne fibres concentration that exceeds the WHO threshold of attention for outdoor ambient (WHO, 2000).

Nevertheless, insufficient attention was devoted to waterborne asbestos analysis in the past, as deeply discussed above. For this reason, available methodological procedures concerning waterborne asbestos analysis are scarce and fragmented. In Italy, no limits are set for asbestos in water and methodologies to asses waterborne asbestos concentration are not shared by all Italian regions.

As previously reported, the guidelines indicated by the Italian U.RP.M842 operating method (ARPA Piemonte, 2016; 2021) were followed in this study. To the authors knowledge, this operating protocol (in the two revisions) is the only one existing which applies to general water samples, not just drinking ones.

As a matter of fact, it is evident from the data reported (see Table 2) that the three different data sets obtained on the same sample do not show good reproducibility among each other. This means that different analytical setups used, the sample coating and interpretation of the operator performing the analysis could influence the final concentration results. This would mean ambiguous results in term of risk assessment with consequent ambiguity in the way of managing the samples. It is necessary to be aware of this and, concerning asbestos, the worst-case scenario should always be considered and action taken accordingly.

Existing round-robin circuits allow to check the interlaboratory reproducibility of a measure, particularly on air samples. On the contrary, too little has been defined at present about waterborne fibres in term of regulatory aspects. Moreover, inter laboratory circuits regarding water samples are not led by supervisory authorities in Italy. So, it is not easy to define whether the misfit among the data sets is mainly related to the analytical setup and method used or to the operator preforming the analysis.

**FIGURE 2:**
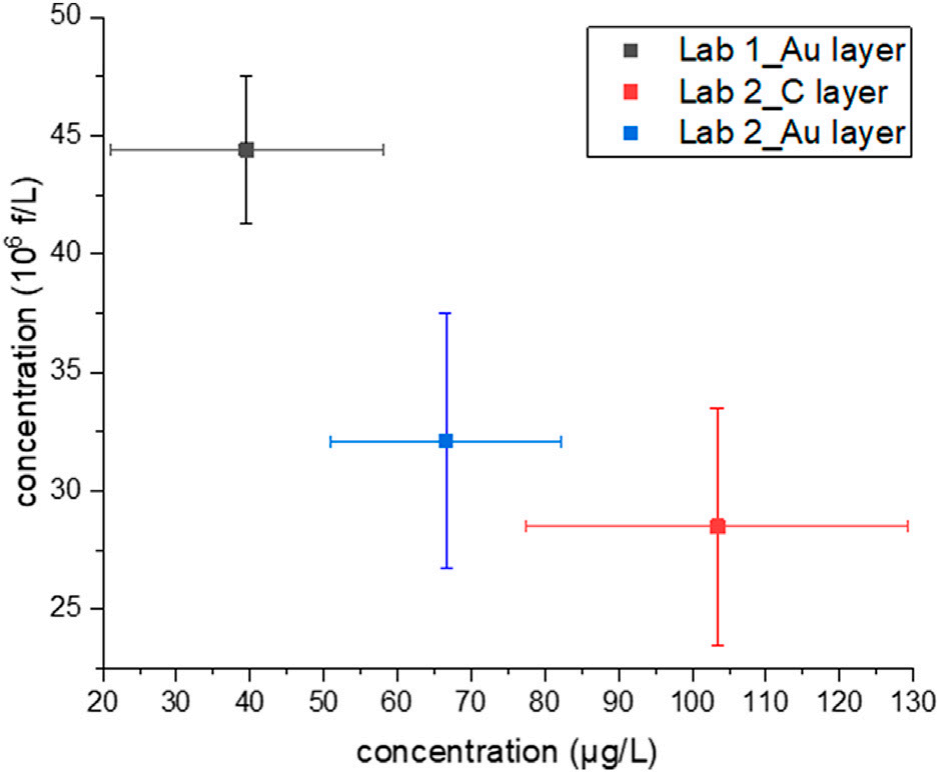
Concentration values in mass (µg/L) vs. concentration in number of fibres per litre (10^6^ f/L) found in the analytical sample using three different setups. Bars represent the 95% confidence limits. Only respirable fibres are considered.

Concerning concentration data reported in this study (see Table 2; Figure 2), produced by different analytical setups and different operators, all confirmed that the sample is in high water contamination range. The concentration, calculated both in f/L and µg/L, is strictly related to the amount of chrysotile originally dispersed in water and a trend could be seen, also considering data reported in Supplementary Table S1. Despite that, more data would be needed to define a precise mathematical relation.

Focussing on data reported in Table 2, it is evident that the reproducibility among different data sets (Lab 1_Au layer, Lab 2_C layer, Lab 2_Au layer) is better if all fibres are counted. When only respirable fibres are counted, the concentration in mass considering the 95% confidence limit provides more overlapping results among the three setups, as shown in Figure 2.

Considering both the concentration in number and mass per litre, the best experimental results could be considered those obtained by Lab 2 analysing an Au coated sample. Indeed, for this sample mid-concentration values were obtained in all categories, partially overlapping with concentration data obtained by Lab 1_Au layer and Lab 2_C layer (Figure 2). This suggests that this is the most reliable data set. For Lab 1_Au layer and Lab 2_C layer, a concentration overestimation or underestimation in mass or number per litre probably occurred.

Furthermore, it is difficult to define a correlation between the concentration in number and mass found. Surprisingly, it would appear that the higher is the content in f/L, the lower is the corresponding concentration in µg/L (Figure 2). All these ambiguities depend primarily on waterborne chrysotile characteristics, such as the degree of defibrillation in water which is strongly related to the power and time of water action on bundles. In addition, some analytical steps in the method are subjected to different interpretations, leading to possibly biased results. Also, minimal instruments requirements have to be defined to guarantee reliable results. For this study, since it is not possible to recognise a trend among the measurements, it is possible to assume that a repeated analytical error is not occurring.

The first important analytical condition to consider is magnification, which could greatly influence the count and the detected particle size (Vigliaturo et al., 2020). Indeed, higher magnification would permit to better visualize details but, maybe, not the entire fibre (particularly when long and thin fibres are present), thus forcing the operator to count it as ½ fibre (see counting criteria, paragraph 2.3). On the contrary, working at lower magnification would permit to see long fibres on the whole but losing very thin ones.

Thus, concerning methodologies, it would be better to indicate a minimum resolution power rather than a magnification. A resolution of 20 pixels/µm, yielding to see details down to 0.050 µm, is necessary to clearly see fibres having width ≥0.1 µm, because at least two adjacent pixel rows have to be recognised to univocally mark the presence of a fibre.

In addition, as a guideline, the authors recommend that rather than counting a fibre partially outside the micrograph as ½ fibre, it would be better to count it as 1 and record its dimensions, avoiding a double counting when the next micrograph is acquired.

**FIGURE 3:**
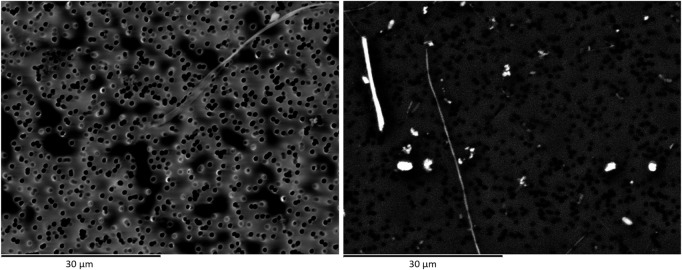
Secondary electron images of two different portions of the same membrane filter. Comparison between Au-layer (left image) and C-layer (right image) coating.

From the data reported in Table 1 concerning image resolution and dimension of minimum visible details, it is clear that using routine SEM with W filament would provide medium quality images, generally permitting to see fibres down to 0.1 µm in width. Then, comparing the three datasets (Table 2) and particle size distributions reported in Figure 1, it emerges that the choice of coating the sample with C or Au layer plays a crucial role in image quality. As shown in Figure 1, it has been possible to detect thinner fibres on samples coated by Au layer (Lab 1_Au layer and Lab 2_Au layer) and concentration values in f/L for those two samples have to be considered more reliable. The use of Au to coat the membranes provides samples with higher conductivity, which translates in images that would be far better resolved than those obtained on samples coated by C layer. As shown in Figure 3, images obtained on Au-coated samples provide better detail comprehension (e.g. fibre bundle edges are sharper than on C-coated sample) even if these images are less contrasted. This influences the image readability (i.e. the definition of details) more than the theoretical resolution of the detector coupled to the SEM (Figure 3).

**FIGURE 4:**
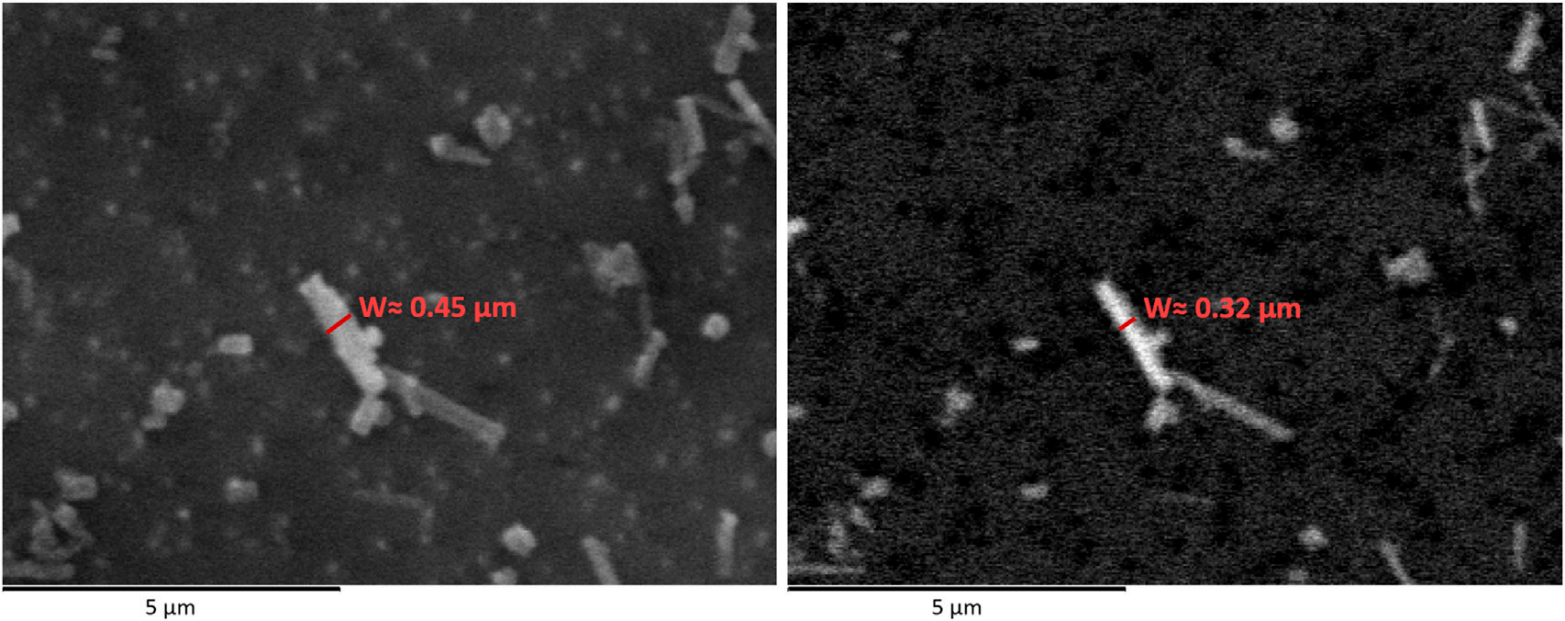
Comparison between a SE (left) and BSE micrograph (right) of the same elongated particle. On both images, the measured particle width is reported.

Another important aspect to evaluate regarding image quality, is to define whether is better to analyse secondary electrons (SE) or backscattered electrons (BSE) images. As already mentioned, no guidelines are reported concerning this topic in the protocols followed (ARPA Piemonte, 2016; 2021). SE images are better in terms of morphology. In BSE images, the brightness and contrast setting can greatly affect the fibre shape, creating an artefact which is larger or smaller than reality and then leading to dimension miscalculation (see Figure 4). Concerning mass calculation, as waterborne fibres are generally very thin, a measuring error of few nanometres multiplied for millions of fibres could result in a miscalculation greater than 20%. It is necessary to keep in mind that concentration values are calculated on the basis of what is seen on a small filter portion and then it is amplified by multiplying it for the total filter area.

Therefore, this has to be considered depending on how resolved are the images and how measuring is made (e.g. on SE or BSE micrographs). In the authors opinion, it would be recommended to count the fibres and measure their dimension on SE images, rather than on BSE ones.

In addition to analytical conditions and instrumental aspects, also the characteristics of the membrane filter itself could affect the final result. Small differences in particle density on the membrane filter could occur, particularly when particles are present in low concentration. For this reason, it is generally recommended to analyse a large area (1 mm^2^) of the filtering surface. In the case presented here, a smaller area was observed because of the high fibre density (ARPA Piemonte, 2021). Checking the homogeneity of particles distribution at low magnification and counting at least 100 fibres, should provide statistically sound data. Therefore, this should not greatly influence the final result. Nevertheless, this might be responsible for the discrepancy in term of concentration in f/L occurred among the two datasets obtained on Au coated samples (Lab 1_Au layer and Lab 2_Au layer).

The presence of bundles or aggregates can greatly influence the concentration results and can generate errors in mass calculation, primarily because it is difficult to define their thickness and three-dimensional structure. Concentration in µg/L found for Lab 2_Au layer and Lab 2_C layer (see Table 2; Figure 2), are higher than the value found for sample Lab 1_Au layer, most likely due to the number of bundles detected (four for each Lab 2 sample). Indeed, bundles greatly affect the mass calculation, while they less affect the count, particularly whether they are compact. On the contrary, the abundant presence of thin fibres, as for sample Lab 1_Au layer, increases the concentration in number while the mass remains the same. Since the analysis can stop when 100 fibres are detected or 20 microscopic fields are observed, in sample Lab 1_Au layer an underestimation of mass concentration probably occurred because mainly thin fibres were detected in the observed area.

Therefore, in term of fibres distribution on the membrane surface, it would be fundamental to better define how to manage with fibre bundles and aggregates, which are difficult to measure and count. It would be anyway important to record on the worksheet how many of those are found.

Concerning aggregates, the authors would recommend as a guideline that the operator counts as many fibres as it is possible to distinguish. If it is not possible to measure all fibres separately, it would be recommended to calculate an average mass based on dimension of those measurable fibres in the aggregate and multiply it by the number of fibres composing the aggregate.

Regarding bundles, the authors support the U.RP.M842 operating method rev. 05 (ARPA Piemonte, 2021) which reports the necessity to consider them in the count, even if the width is larger than 3 µm (despite the respirable counting criteria) (WHO, 1986). Indeed, they are a potential source of a great quantity of thinner fibres if further disaggregation occurs in water. A compact bundle could be counted as one unit and mass can be considered on the whole, measuring width in the central compact part of the bundle and length excluding the fringed ends. In case of bundles with ends that open up in thinner fibres, they could be counted as a number of fibres equal to those whose both ends are visible. Regarding mass calculation, it would be better to ideally divide it into compact subunits to measure separately.

In the opposite case, when many thin fibres are present, the measurement could be very time consuming. Therefore, it would be recommendable to only measure their length and give them an arbitrary width equal to the minimum width clearly recognisable on micrographs (e.g. 0.1 µm).

In light of the above, it is strongly recommended that the analysts performing this type of analyses are specifically formed. In addition, there exist some confounding factors that they should be aware of. One of this is the presence of biomineralizations containing Si having a fibre-like shape, which are quite common in surface water samples (Bruno M.R., personal communication, 20 September 2022). Only an attentive evaluation of EDS spectra could discriminate those from asbestos fibres. Therefore, it is important to carefully consider the morphology together with the chemical data in order to define what is asbestos.

Since hazard related to ingestion of asbestos fibres is still unclear (WHO, 2021), the principal assessment measure taken in account at present would be the possible migration to air. In this scenario, it is clear that waterborne fibres falling in the respirable range (WHO, 1986) have to be considered as potentially dangerous. Therefore, the authors strongly believe that all fibres longer than 5 µm should be considered for the final concentration evaluation, unlike what is required by US-EPA method (US-EPA, 1994). In addition, it would be useful to record the presence of shorter fibres.

A concentration in number of fibres per litre is fundamental to express because it can be directly correlated to the number of fibres which can possibly migrate to air (Roccaro and Vagliasindi, 2018; Avataneo et al., 2022). Further investigation is needed to better understand why millions of waterborne fibres yield a few units in air, although these airborne fibres are above the attention threshold (WHO, 2000).

Considering concentration in mass per litre, it is shown in Table 2 that the concentration found in water considering fibres with L > 5 µm is lower than the nominal concentration calculated based on the amount initially dispersed in water (137.40 μg/L). The standard chrysotile dispersed in water is partially composed by shorter fibres (length ≤5 µm) that are not counted under the adopted analytical method and, during water motion, longer and thicker bundles may undergo defibrillation achieving shorter lengths (L ≤ 5 µm) (Avataneo et al., 2022).

The calculated value is indeed closer to the nominal value when all fibres are considered (see Table 2), but still there is a discrepancy among those data. This could derive from a partial deposition of large bundles during the water motion process or to adhesion of fibres on tank walls, possibly induced by electrostatic forces. Nevertheless, the powder dispersed in water to prepare the analytical sample could contain traces of mineral species other than chrysotile (Fornasini et al., 2022) that would not be counted during the analysis, thus not contributing to the final mass calculation. Finally, a miscalculation due to the fact that many fibres lie partially outside of the images acquired (see counting criteria, paragraph 2.3) on which measurements are performed could also influence the final concentration value.

To date, the concentration expression in µg/L for water samples containing asbestos is not required by the Italian control organs. They only recommend the expression in f/L, which is in closer relation to the number of fibres that can eventually migrate to air. Conversely, we strongly believe that the expression in mass per litre would be equally important because it would give a hint on the asbestos mass dispersed in water, more closely related to the pollution source (NOA or man-made asbestos containing materials).

In term of exposure assessment, we believe that the concentration in mass per litre would give more representative information regarding the level of water contamination, since the number would instead depend also on the time and power of water motion which can translate into defibrillation and disaggregation of large bundles.

In case a linear relation is seen between the concentration in mass and number, a fast conversion from number of fibres to mass would be possible and certainly time-saving. This conversion would be possible only in case that waterborne fibres are classed in dimension (Avataneo et al., 2020). This is an unlikely scenario since different average masses can even be calculated considering fibres found in different portions of the same filter membrane, as it is shown in Table 2.

As deeply discussed in this study, many analytical ambiguities have to be addressed by the control organs to achieve sufficiently sound data about waterborne asbestos concentrations, particularly regarding non-drinking water investigations. Analytical procedures standardization is urgently needed to improve the reliability of waterborne measurements (Turci et al., 2016) and also to define a shared method among different countries.

The necessity to perform waterborne asbestos analyses by means of TEM, as required from North American regulations (US-EPA, 1994) or SEM has to be address. Since this study is set in Italy, the authors opted for SEM-EDS investigations, as required by Italian analytical methods already presented (ARPA Piemonte, 2016; 2021; Italian National Institute of Health, 2016). In addition, samples preparation for TEM-EDS analysis and analysis itself are more difficult, time-consuming and expensive than those for SEM-EDS.

Furthermore, based on the width ranges presented in this study (see Figure 1), the magnification power of the SEM was considered suitable for fibres analyses. A different situation could occur when groundwater samples are analysed, since in this case waterborne fibres show width down to few tens on nanometres (Avataneo et al., 2021) and, thus, TEM analysis is required.

Lastly, novel techniques that can be applied to waterborne asbestos investigations have to be evaluated by the control organs, to perform faster and more reliable analyses. Particularly, the unattended SEM-EDS analysis proposed by Cossio et al. (2018) would allow a faster analysis providing results not influenced by the analyst. A novel technique by Li et al. (2019) using Phase Contrast Microscopy (PCM) coupled with micro-Attenuated Total Reflectance coupled to Fourier Transform Infrared spectroscopy (µ-ATR-FTIR), would allow a quick detection of asbestos in drinking waters, suitable for water screening for fibres having length >10 µm because of spatial resolution limits. Also, the *in-situ liquid cell spinning-disk confocal microscopy* (Wu et al., 2015) is an innovative fluorescence-microscopy method which possibly allows to perform automated counting and to detect in real time the Brownian motion of waterborne asbestos.

The setting of the minimum instrumental requirement for water investigations and the definition of standardized analytical method, together with the validation of innovative techniques, are crucial to define efficient analytical methods for waterborne asbestos investigations. This is needed to lead a reliable water contamination assessment and, therefore, to assess environmental risk and reduce population exposure. These are fundamental steps to define a shared MCL of asbestos in water (particularly regarding non-drinking water).

## 5 Conclusion

This study was designed to drive the attention of environmental protection agencies on the evaluation of analytical protocols available for waterborne asbestos analyses, particularly referring to the Italian legislation and dealing with SEM analyses of surface waters.

Three data sets were obtained on the same asbestos-containing water sample, using different analytical setups and with analysis performed by different operators. A comparison among the data sets has been made to highlight that good interlaboratory reproducibility is not guaranteed using the methods available at present in Italy and to discuss which could be the most error-prone analytical steps.

It has been underlined that inter laboratory circuits and minimal instrumentation requirements have to be defined at the earliest opportunity by the regulatory organs to improve the results representativeness. The type of instrumental set up and analytical conditions used for the analyses could influence the result. Also, the necessity of better counting rules definition has been addressed, mainly in case of bundles or aggregates occurrence. In addition, it would be necessary to define whether just respirable fibres have to be counted in water.

Lastly, we dealt with the necessity to express the waterborne asbestos concentration in µg/L, that could be useful to better define the water contamination level.

All these aspects have been discussed in detail and possible implementation and improvements in methodologies have been proposed in order to help the regulatory organs to better define analytical protocols. The definition of a detailed method is urgent for waterborne fibres analyses, particularly referring to surface water which could create a dangerous situation for the environment and the local population in NOA-rich areas.

Concerning groundwater contamination by asbestos (or other mineral fibres non-asbestos classified) in NOA-rich settings, the authors believe that further studies have to be done to better define a dedicated analytical method, possibly involving TEM-EDS investigations.

All these aspects are preparatory to the definition of a Maximum Contaminant Level for waterborne asbestos shared among all countries.

## Data Availability

The original contributions presented in the study are included in the article/Supplementary material, further inquiries can be directed to the corresponding author.

## Footnotes

1
http://www.rsa-srl.it/

## References

[B1] Anastasiadou K. Gidarakos E. (2007). Toxicity evaluation for the broad area of the asbestos mine of northern Greece. J. Hazard. Mater. 139 (1), 9–18. 10.1016/j.jhazmat.2006.06.031 16889894

[B2] ANSES (2017). Anses-Agence Nationale de Sécurité Sanitaire de l’Alimentation de l’Environnement et du Travail. Avis de l’Anses Saisine n. 2016-SA-0034 relatif aux « Particules minérales allongées. Identification des sources d’émission et proposition de protocoles de caractérisation et de mesures. (in French) https://www.anses.fr/fr/system/files/AIR2016SA0034Ra.pdf (Accessed October 10, 2022).

[B3] ANSI (2009). Ansi-American National Standards Institute, D6281-09, Standard test method for airborne asbestos concentration in ambient and indoor atmospheres as determined by transmission electron microscopy direct transfer (TEM).

[B4] ARPA Piemonte (2016). Piedmont Environmental Protection Agency (Agenzia regionale per la protezione ambientale). U.RP.M842 rev.03. Asbestos in water by Scanning Electron Microscopy. (Amianto in acqua in Microscopia Elettronica a Scansione, in Italian). Available at: https://www.regione.piemonte.it/web/sites/default/files/media/documenti/2019-06/7.Allegato%207_%20Metodo%20ARPA%20Piemonte%20_Amianto%20in%20acqua.pdf .

[B5] ARPA Piemonte (2021). Piedmont Environmental Protection Agency (Agenzia regionale per la protezione ambientale). U.RP.M842 rev.05. Asbestos in water by Scanning Electron Microscopy. (Amianto in acqua in Microscopia Elettronica a Scansione, in Italian).

[B6] Avataneo C. Belluso E. Bergamini M. Capella S. De Luca D. A. Lasagna M. (2020). Waterborne naturally occurring asbestos: A case study from Piedmont (NW Italy) EGU General Assem. Online, 4–8 May 2020, EGU2020- 19615. 10.5194/egusphere-egu2020-19615

[B7] Avataneo C. Belluso E. Capella S. Cocca D. Lasagna M. Pigozzi G. (2021). Groundwater asbestos pollution from naturally occurring asbestos (NOA): A preliminary study on the lanzo valleys and Balangero plain area, NW Italy. Italian J. Eng. Geol. Environ. 1, 5–19. 10.4408/IJEGE.2021-01.S-01

[B8] Avataneo C. Petriglieri J. R. Capella S. Tomatis M. Luiso M. Marangoni G. (2022). Chrysotile asbestos migration in air from contaminated water: An experimental simulation. J. Hazard. Mater. 424, 127528. 10.1016/j.jhazmat.2021.127528 34736189

[B9] Bailey R. M. (2020). Asbestiform minerals of the franciscan assemblage in California with a focus on the calaveras dam replacement project. Environ. Eng. Geoscience 26 (1), 21–28. 10.2113/EEG-2264

[B10] Belluso E. Baronnet A. Capella S. (2020). Naturally occurring asbestiform minerals in Italian Western alps and in other Italian sites. Environ. Eng. Geoscience 26 (1), 39–46. 10.2113/EEG-2276

[B11] Berry T.-A. Belluso E. Vigliaturo R. Gieré R. Emmett E. A. Testa J. R. (2022). Asbestos and other hazardous fibrous minerals: Potential exposure pathways and associated health risks. Int. J. Environ. Res. Public Health 19, 4031. 10.3390/ijerph19074031 35409711 PMC8998304

[B12] Bloise A. Ricchiuti C. Giorno E. Fuoco I. Zumpano P. Miriello D. (2019). Assessment of naturally occurring asbestos in the area of Episcopia (Lucania, Southern Italy). Fibers 7 (5), 45. 10.3390/fib7050045

[B13] Botta S. Avataneo C. Barale L. Compagnoni R. Cossio R. Marcelli I. (2020). Petrofacies for the prediction of NOA content in rocks: Application to the “gronda di Genova” tunneling project. Bull. Eng. Geol. Environ. 79 (1), 185–204. 10.1007/s10064-019-01539-6

[B14] Buck B. J. Goossens D. Metcalf R. V. McLaurin B. Ren M. Freudenberger F. (2013). Naturally occurring asbestos: Potential for human exposure, southern Nevada, USA. Soil Sci. Soc. Am. J. 77 (6), 2192–2204. 10.2136/sssaj2013.05.0183

[B15] Buzio S. Pesando G. Zuppi G. M. (2000). Hydrogeological study on the presence of asbestos fibres in water of northern Italy. Water Res. 34 (6), 1817–1822. 10.1016/S0043-1354(99)00336-X

[B16] Cossio R. Albonico C. Zanella A. Fraterrigo-Garofalo S. Avataneo C. Compagnoni R. (2018). Innovative unattended SEM-EDS analysis for asbestos fiber quantification. Talanta 190, 158–166. 10.1016/j.talanta.2018.07.083 30172493

[B17] DM 06/09/1994 - Ministerial Decree (Decreto Ministeriale) (1994). Regulations and technical methodologies for the application of article 6-paragraph 3 and article 12-paragraph 2 of the law n. 257, 27 March 1992, referring to the termination of use of asbestos. (in Italian). Available at: https://www.gazzettaufficiale.it/eli/id/1994/09/20/094A5917/sg (Accessed November 3, 2022).

[B18] Erskine B. G. Bailey M. (2018). Characterization of asbestiform glaucophane-winchite in the franciscan complex blueschist, northern diablo range, California. Toxicol. Appl. Pharmacol. 361, 3–13. 10.1016/j.taap.2018.09.020 30240695

[B19] European Parliament and Council (2020). Directive 2020/2184 of 16 december 2020 on the quality of water intended for human consumption. https://eur-lex.europa.eu/eli/dir/2020/2184/oj.

[B20] Fornasini L. Raneri S. Bersani D. Mantovani L. Scognamiglio V. Di Giuseppe D. (2022). Identification of iron compounds in chrysotile from the Balangero mine (Turin, Italy) by micro-Raman spectroscopy. J. Raman Spectrosc. 1, 1931–1941. 10.1002/jrs.6434

[B21] IARC (2012). International Agency for Research on Cancer. Arsenic, metals, fibres, anddusts, Monograph on the evaluation of carcinogenic risks To humans. http://monographs.iarc.fr/ENG/Monographs/vol100C/mono100C.pdf.

[B22] IBAS (2022). International Ban Asbestos Secretariat. Revised 14 july 2022. http://ibasecretariat.org/alpha_ban_list.php# (Accessed November 2, 2022).

[B26] ISO (2014). International Organization for Standardization. ISO 8672:2014 Air quality - determination of the number concentration of airborne inorganic fibres by phase contrast optical microscopy - membrane filter method. Geneva, Switzerland: ISO.

[B24] ISO (2019a). International Organization for Standardization. ISO 10312:1995 (revised in 2019) Ambient air – determination of asbestos fibres - direct-transfer transmission electron microscopy method. Geneva, Switzerland: ISO.

[B23] ISO (2019b). International Organization for Standardization. ISO 13794:1999 (revised in 2019) Ambient air – determination of asbestos fibres - indirect-transfer transmission electron microscopy method. Geneva, Switzerland: ISO.

[B25] ISO (2019c). International Organization for Standardization. ISO 14966:2002 (revised in 2019) Ambient air – determination of numerical concentration of inorganic fibrous particles- Scanning electron microscopy method. Geneva, Switzerland: ISO.

[B27] ISS (2016). Italian National Institute of Health (Istituto Superiore di Sanità) “ISS.EAA.000 Analytical method for qualitative and quantitative determination of asbestos fibres concentration in drinking water with Scanning Electron Microscopy (SEM) technique,” in Italian Ministry of health. Asbestos: Summary of knowledge relating to exposure and toxicological profile. Available at: https://www.salute.gov.it/portale/temi/documenti/acquepotabili/parametri/Val_Amianto_documento_completo.pdf (Accessed November 2, 2022).

[B28] Jacques O. Pienitz R. (2022). Assessment of asbestos fiber contamination in lake sediment cores of the Thetford Mines region, southern Quebec (Canada). Environ. Adv. 8, 100232. 10.1016/j.envadv.2022.100232

[B29] Kashansky S. V. Slyshkina T. V. (2002). Asbestos in water sources of the Bazhenovskoye chrysotile asbestos deposit. Int. J. Occup. Med. Environ. Health. 15, 65–68. 12038867

[B30] Koumantakis E. Kalliopi A. Dimitrios K. Gidarakos E. (2009). Asbestos pollution in an inactive mine: Determination of asbestos fibers in the deposit tailings and water. J. Hazard. Mater. 167, 1080–1088. 10.1016/j.jhazmat.2009.01.102 19304382

[B31] Li J. Li H. Zheng B. Yu Z. (2019). Comparison of analysis of asbestos fibres in drinking water using phase contrast microscopy and micro-FTIR spectrometry with scanning electron microscopy and energy-dispersive X-ray spectroscopy. Environ. Sci. Water Res. Technol. 5, 543–551. 10.1039/c8ew00863a

[B32] Malinconico S. Paglietti F. Serranti S. Bonifazi G. Lonigro I. (2022). Asbestos in soil and water: A review of analytical techniques and methods. J. Hazard. Mater. 436, 129083. 10.1016/j.jhazmat.2022.129083 35576665

[B33] Meranger J. C. Reid W. W. Davey A. B. C. (1979). The transfer of asbestos from water to air via a portable drum-type home humidifier. Can. J. Public Health 70, 276–278. 526901

[B34] Mohanty S. K. Salamatipour A. Willenbring J. K. (2021). Mobility of asbestos fibers below ground is enhanced by dissolved organic matter from soil amendments. J. Hazard. Mater. Lett. 2, 100015. 10.1016/j.hazl.2021.100015

[B36] NIOSH (1994). National Institute for Occupational Safety and Health. Method 7402 - asbestos by TEM, in NIOSH manual of analytical methods (NMAM). 4th edn. https://www.cdc.gov/niosh/docs/2003-154/pdfs/7402.pdf (Accessed October 5, 2022).Method 7402 asbestos by TEM.

[B35] NIOSH (2019). National Institute for Occupational Safety and Health. Method 7400 - asbestos and other fibers by PCM, in NIOSH manual of analytical methods (NMAM). 5th edn. https://www.cdc.gov/niosh/docs/2003-154/pdfs/7400.pdf (Accessed October 5, 2022).

[B37] Roccaro P. Vagliasindi F. G. A. (2018). Indoor release of asbestiform fibers from naturally contaminated water and related health risk. Chemosphere 202, 76–84. 10.1016/j.chemosphere.2018.03.040 29554510

[B38] RSA (2019). Public company in charge of the remediation and environmental development of the former asbestos mine site of Balangero and Corio municipalities. Environvental monitoring report 2018-2019 (Rapporto Monitoraggio Ambientale 2018-2019, in Italian). http://www.rsa-srl.it/RapportoMonitoraggioAmbientaleAnno2018_19.pdf (Accessed September 23, 2022).

[B39] Schreier H. (1989). Studies in environmental science, Asbestos in the natural environment, 37. The Netherlands: Elsevier Science Publisher B.V.

[B40] Thives L. P. Ghisi E. Júnior J. J. T. Vieira A. S. (2022). Is asbestos still a problem in the world? A current review. J. Environ. Manag. 319, 115716. 10.1016/j.jenvman.2022.115716 35863303

[B41] Turci F. Favero-Longo S. E. Gazzano C. Tomatis M. Gentile-Garofalo L. Bergamini M. (2016). Assessment of asbestos exposure during a simulated agricultural activity in the proximity of the former asbestos mine of Balangero, Italy. Italy. J. Hazard. Mater. 308, 321–327. 10.1016/j.jhazmat.2016.01.056 26852207

[B42] US-EPA (1994). States Environmental Protection Agency. Method 100.2 Determination of Asbestos structures over 10 μm in length in drinking water . EPA/600/R-94/134. https://nepis.epa.gov/ .

[B43] US-EPA (2004). United States Environmental Protection Agency. Safe drinking water act. EPA 816-F-04-030. https://www.epa.gov/ground-water-and-drinking-water/national-primary-drinking-water-regulations#Inorganic (Accessed November 18, 2022).

[B44] Vigliaturo R. Choi J. K. Pérez-Rodríguez I. Gieré R. (2020). Dimensional distribution control of elongate mineral particles for their use in biological assays. MethodsX 7, 100937. 10.1016/j.mex.2020.100937 32566490 PMC7298544

[B45] Webber J. S. Syrotynski S. King M. V. (1988). Asbestos-contaminated drinking water: Its impact on household air. Environ. Res. 46, 153–167. 10.1016/s0013-9351(88)80029-x 3402404

[B48] WHO (1986). World Health Organization. Asbestos and other natural mineral fibres. Environmental Health Criteria No. 53. Geneva, Switzerland: World Health Organization.

[B47] WHO (2000). World Health Organization. Air quality guidelines for Europe. 2nd edn.Copenhagen, Denmark European series: WHO regional publications. No. 91. 11372513

[B46] WHO (2021). World Health Organization. Asbestos in drinking-water. Background document for development of WHO Guidelines for drinking-water quality. WHO/HEP./ECH/WSH/2021.4. Geneva, Switzerland: World Health Organization.

[B49] Wu L. Ortiz C. Xu Y. Willenbring J. Jerolmack D. (2015). *In situ* liquid cell observations of asbestos fiber diffusion in water. Environ. Sci. Technol. 49 (22), 13340–13349. 10.1021/acs.est.5b03839 26461183 PMC4747642

