# Supplementary material for: Waterborne asbestos: Good practices for surface waters analyses
Source: Front Chem. 2023 Jan 25;11:1104569. doi: 10.3389/fchem.2023.1104569 (PMC9905445; doi:10.3389/fchem.2023.1104569)
Supplement: Supplementary file 1 [file Table1.DOCX]

# Supplementary material

**Table S1: experimental data obtained on samples containing nil (Tap water), low and mid waterborne chrysotile, analysed using different setups (Lab 1_Au layer, Lab 2_C layer). The three samples were prepared *ad hoc* in laboratory by adding selected chrysotile powder from Balangero former mine (Italy) to tap water. The confidence limits (LFL-UFL and ΔC) are reported for each sample.**

|  | Lab 1_Au layer | | | | | Lab 2_C layer | | | | |
| --- | --- | --- | --- | --- | --- | --- | --- | --- | --- | --- |
| Sample name | Concentration [f/L] | LFL | UFL | Concentration [µg/L] | ΔC [µg/L] | Concentration [f/L] | LFL | UFL | Concentration [µg/L] | ΔC [µg/L] |
| Tap water | **<LOD** | - | - | **<LOD** | **-** | **<LOD** | - | - | **<LOD** | - |
| Low chrysotile concentration | **3.70·10^6^** | 3.41·10^6^ | 4.02·10^6^ | **4.10** | 1.33 | **1.34·10^6^** | 6.22·10^5^ | 2.58·10^6^ | **3.50** | 2.59 |
| Mid chrysotile concentration | **1.11·10^7^** | 1.04·10^7^ | 1.20·10^7^ | **4.57** | 0.92 | **2.24·10^6^** | 1.34·10^6^ | 3.71·10^6^ | **5.84** | 1.73 |
